# Potential of Chitosan-Based Basil Essential Oil Nanoparticles in Preventing Microbial Contamination of Beef

**DOI:** 10.3390/antibiotics15050442

**Published:** 2026-04-29

**Authors:** Natalija Đorđević, Ivana Karabegović, Jelena Stanojević, Pero Sailović, Slavica Vesković Moračanin, Dragiša Savić, Bojana Danilović

**Affiliations:** 1 Faculty of Technology, University of Niš, Bulevar Oslobođenja 124, 16000 Leskovac, Serbia; natalija@tf.ni.ac.rs (N.Đ.); ivana.karabegovic@tf.ni.ac.rs (I.K.); jstanojevic@tf.ni.ac.rs (J.S.); savic@tf.ni.ac.rs (D.S.); 2 Faculty of Technology, University of Banja Luka, Bulevar Vojvode Stepe Stepanovića 73, 78000 Banja Luka, Bosnia and Herzegovina; pero.sailovic@tf.unibl.org; 3 Institute of Meat Hygiene and Technology, Kaćanskog 13, 11000 Belgrade, Serbia; slavica.veskovic@inmes.rs

**Keywords:** basil essential oil, beef storage, chitosan coating, nanoparticles

## Abstract

Background: Microbial contamination of fresh beef remains a major challenge in the meat industry, driving the need for effective natural preservation strategies that can extend shelf life while meeting consumer demand. Methods: Chitosan-based edible coatings enriched with free and nanoencapsulated *Ocimum basilicum* L. essential oil at concentrations of 0.25%, 0.5% and 1% were evaluated for their efficacy on fresh beef during 20 days of refrigerated storage. Microbiological parameters, including total bacterial count, lactic acid bacteria, psychrotrophic bacteria, and *Pseudomonas* spp., as well as physicochemical indicators such as pH and thiobarbituric acid reactive substances, were monitored at regular intervals throughout storage. Results: All active coatings significantly retarded microbial growth and lipid oxidation compared to the uncoated control (*p* < 0.05), with effects being concentration-dependent. Nanoencapsulation was achieved with an efficiency of 74%, and all formulations consistently showed better results compared to free essential oil coatings at equivalent concentrations. Application of a chitosan coating with 1% nanoencapsulated essential oil reduced total viable count by 1.5 log CFU/g and lactic acid bacteria by 0.7 log CFU/g, with the most pronounced effect observed for *Pseudomonas* spp. (1.9 log CFU/g reduction). In the same sample, MDA content remained below the threshold level until the end of storage. Additionally, sensory analysis indicated that the use of nanoparticles significantly improved the overall acceptability of the coated beef. Conclusions: These findings confirm that chitosan–basil nanoparticle coatings represent a promising natural alternative to conventional preservatives for improving microbiological safety and extending the shelf life of fresh beef.

## 1. Introduction

Meat and meat products are among the most perishable food commodities, with their shelf life and safety largely determined by the initial microbial load, preservation method, packaging type, and storage conditions. Inadequate handling along the supply chain can result in significant economic losses for the meat industry, reaching up to 40% of total production, while simultaneously posing significant public health risks [1]. The search for effective, natural, and consumer-friendly preservation strategies has therefore become one of the central challenges in modern food technology.

Essential oils derived from aromatic plants have attracted considerable attention as natural antimicrobial agents with broad-spectrum activity. Among these, basil (*Ocimum basilicum* L.) a perennial herbaceous plant from the family *Lamiaceae*, widely distributed across tropical and subtropical regions of Asia, Africa, and the Americas, stands out as a particularly well-studied source of bioactive compounds. Its phytochemical profile is dominated by secondary metabolites, primarily flavonoids and terpenoids, which are widely considered to contribute to its therapeutic and antimicrobial potential [2]. The principal components of *O. basilicum* essential oil (OBEO) typically belong to the monoterpene alcohols, phenylpropanoids, and oxygenated monoterpenes [3,4]. Linalool, a monoterpene alcohol responsible for the oil’s characteristic floral note, has been shown to exhibit antimicrobial, antioxidant, and anti-inflammatory properties [5]. Other notable constituents such as *p*-cymene, cis-sabinene hydrate, and estragole have been investigated for their antioxidant, anti-inflammatory, and even antitumor activities, with emerging applications in both medicine and food packaging [6,7,8].

Despite this promising biological profile, the practical application of essential oils in food preservation is substantially limited by their volatility and sensitivity to environmental factors such as light, oxygen, moisture, and temperature. Under processing and storage conditions, thermally and oxidatively unstable components can degrade rapidly, forming potentially harmful by-products and losing their antimicrobial efficacy [9,10]. Furthermore, when incorporated directly into food matrices, essential oils tend to bind to carbohydrates, proteins, or lipids, which further reduces their bioavailability and forces the use of higher concentrations, with predictable negative consequences for the sensory properties of the final product [11].

Nanoencapsulation represents a promising approach for addressing these limitations. By enclosing essential oils within nanocarriers, most commonly biopolymer-based nanoparticles, it is possible to minimize the impact of external factors, prevent the loss of volatile components, improve solubility and bioavailability, and achieve controlled, targeted release of bioactive compounds within biological or food systems [12,13,14]. An additional practical advantage of nanoencapsulation is the masking of intense essential oil aromas, which is of particular importance in food applications where consumer acceptance is essential [15].

Chitosan, one of the most abundant biopolymers in nature, is especially well-suited as a nanoparticle matrix due to its biodegradability, non-toxicity, and biocompatibility, alongside favorable antimicrobial properties of its own [16]. The antimicrobial mechanism of chitosan-based nanoparticles is primarily driven by electrostatic interactions between the positively charged amino groups of chitosan and the negatively charged components of microbial cell membranes, leading to membrane disruption and the inhibition of cellular functions [17]. When loaded with essential oils, chitosan nanoparticles present an enhanced biological profile, offering both antimicrobial and antioxidant activities that can prevent microbial spoilage and lipid oxidation, the two principal deteriorative processes in fresh meat [18,19].

The microbial ecology of fresh beef spoilage is complex, involving a succession of microorganisms that vary depending on storage conditions and packaging. *Pseudomonas* spp. are typically the dominant contaminants under aerobic refrigerated conditions, producing biogenic amines and hydrolytic enzymes that lead to off-odors and structural degradation [20,21]. Lactic acid bacteria, while traditionally viewed as beneficial in fermented products, can also cause spoilage under vacuum or modified atmosphere conditions, producing metabolites that alter texture, flavor, and pH [22]. Pathogenic organisms such as *Salmonella* spp., *Listeria monocytogenes*, and *Escherichia coli* pose additional food safety concerns, with *L. monocytogenes* being of particular interest due to its capacity to grow at refrigeration temperatures and survive at low pH and water activity values [23,24].

Given this context, the aim of the present study was to evaluate the potential of chitosan-based nanoparticles loaded with OBEO in inhibiting microbial contamination and extending the shelf life of beef under refrigerated storage conditions.

## 2. Results and Discussion

### 2.1. Nanoparticles Characterization

The results of determining particle size (Z-average), polydispersity index (PDI) and encapsulation efficiency (EE, %) (Table 1) indicated that chitosan nanoparticles loaded with *O. basilicum* L. essential oil (OBNPs) had a smaller average particle size (177.4 nm) compared to pure chitosan nanostructures (415.4 nm). This result suggests that the incorporation of OBEO contributes to a reduction in particle size, probably due to interactions of the hydrophobic components of the oil with chitosan during the nanocapsule formation process, leading to a more compact and homogeneous structure [25]. Similar results have been reported in the literature, where nanoencapsulation of essential oils leads to a reduction in particle size. For example, Đorđević et al. [26] showed that nanoencapsulation of a mixture of essential oils of *Ocimum basilicum* L. and *Satureja montana* L. yields particles in the range of 150–200 nm, with increased stability and biological activity.

A similar effect has been observed in other studies. Namely, Ma et al. [27] obtained chitosan nanoparticles with incorporated oregano essential oil with a size of about 183 nm, while Yousefi et al. [28] reported a range of 198–318 nm for ginger-chitosan nanoparticles. One of the main mechanisms that explains this phenomenon is the so-called shrinkage/compaction effect, whereby essential oil components act as plasticizers within the chitosan matrix, enabling denser packing of polymer chains and more efficient cross-linking with TPP [27]. Additionally, the distribution of essential oil within the polymer matrix network can reduce the effective wall thickness of the nanoparticles, which also contributes to a reduction in their size [28]. Furthermore, the incorporation of essential oil can improve the colloidal stability of the system and reduce the aggregation of particles, which directly reflects on their size [27]. Moreover, the choice of essential oil and its chemical composition, the choice of plasticizer, and the synthesis method have a direct impact on the size of the synthesized nanoparticles [29].

The PDI for OBNPs is 0.221, indicating high uniformity and dispersion stability. Based on the standard classification, PDI values < 0.3 indicate a homogeneous particle size distribution, while values above 0.5 indicate a high degree of heterogeneity [30]. In contrast, chitosan nanoparticles (CHNPs) exhibited a high PDI value (0.718), indicating a heterogeneous size distribution and reduced colloidal stability, which is typically associated with particle aggregation [30,31]. This behavior may be attributed to the absence of hydrophobic components, such as an essential oil, which can act as a structural filler and promote the formation of more compact and uniform nanostructures [25,32]. In the literature, PDI values between 0.5 and 0.8 are often reported for chitosan nanoparticles without an active substance, while the addition of hydrophobic bioactive compounds (essential oils, flavonoids, phenols) usually reduces this value [33].

For OBNPs, an encapsulation efficiency of 73.84 ± 4.63% was obtained, meaning that more than two-thirds of the oil was successfully incorporated into the chitosan matrix. Encapsulation efficiency values above 70% are generally considered satisfactory for phytochemicals and essential oils, due to their complex composition and high volatility, which make their retention within carrier systems challenging [32,34,35]. Comparable EE values have also been reported for OBEO incorporated into chitosan nanoparticles. For instance, an encapsulation efficiency of 75.13 ± 0.09% was achieved at a chitosan/OBEO ratio of 1:0.5 [36]. Additionally, encapsulation efficiency values ranging from 50.39% to 75.13% were observed depending on the chitosan-to-oil ratio, with the highest encapsulation efficiency obtained at a lower oil content. A decrease in encapsulation efficiency at higher oil ratios has been attributed to the saturation of the chitosan matrix, probably limiting further incorporation of the essential oil [37]. In the work of Volić et al. [33], the design of biopolymer carriers for thyme essential oil yielded similar values of encapsulation efficiency (65–80%), while smaller nanodiameters and lower PDI correlated with better encapsulation and a more controlled release.

### 2.2. Changes in Microbial Counts During Beef Storage

#### 2.2.1. Total Viable Count

The results of TVC number in samples of raw beef treated with chitosan coatings with the addition of basil (*Ocimum basilicum* L.) essential oil are presented in Table 2 and indicate a significant impact of the treatment on the microbiological stability of the meat during storage. All samples had a similar initial TVC number, in the range of 2.3–2.6 log CFU/g. In the control sample, the fastest increase in TVC was observed, which was expected given the high water and nutrient content that favors the development of microorganisms in raw meat. Compared to the control, the application of a chitosan coating (CH) caused a statistically significant (*p* < 0.05) reduction in the number of TVC after the fourth storage day, which confirms the antimicrobial effect of chitosan, probably as a consequence of its polycationic nature, which allows interaction with microbial cells and leads to their destabilization [38].

After 8 days of storage, the control sample reached 5.95 log CFU/g, which is very close to the defined regulatory threshold for fresh meat specified in the International Commission on Microbiological Specifications for Foods (ICMSF) standard [39] and the Chinese national standard GB 4789.2-2022 [40], according to which TVC values higher than 6.0 log CFU/g indicate a microbiologically unacceptable product [11]. During the same period, the application of OBEO further reduced the total bacterial count: at a concentration of 0.25% it was 5.15 log CFU/g, at 0.5% it was 5.09 log CFU/g, while at 1% the lowest value of 4.21 log CFU/g was recorded, clearly confirming a pronounced dose-dependent effect. Statistically, the application of nanoparticles at concentrations of 0.5% and 1% had the same effect on the same day of storage.

During further storage, a clear difference between the effects of free CH-OBEO and CH-OBNPs systems on the TVC values was observed. This difference became most pronounced at the end of the storage period, when the statistically significant largest reduction was recorded in the treatment with OB NPs 1% with a value of 6.55 log CFU/g, which represents a decrease of approximately 1.5 log units compared to the control sample. Based on the obtained TVC values, the application of CH-OBEO can significantly extend the microbiological acceptability of beef, with the storage period extending to approximately 16 days compared to the control.

The observed difference in antimicrobial efficacy between free CH-OBEO and CH-OBNPs systems can be explained, most likely, by the physicochemical properties of the nanoparticles, particularly their particle size and polydispersity index (PDI). Smaller particles have a higher surface area, which promotes closer contact with microbial cell membranes and enhances the delivery of bioactive compounds from the essential oils [41]. At the same time, their reduced size facilitates penetration through microbial structures and biofilms, contributing to improved antimicrobial performance [42].

In addition, low PDI values reflect a more uniform particle size distribution, resulting in better dispersion and a more consistent release of the encapsulated essential oil components. Such systems tend to exhibit more stable antimicrobial activity, whereas systems with higher PDI may be less effective due to the presence of larger particles with limited interaction potential and smaller ones that are more prone to aggregation [43,44].

Numerous studies confirm that nanoencapsulation of essential oils is a more effective approach to controlling TVC in meat, compared to the application of free essential oils. In the study of Đorđević et al. [45], chitosan coatings with nanoencapsulated essential oil of *Satureja montana* showed a more pronounced inhibitory effect on microbial growth in beef during 20 days of storage, with a decrease in TVC by several log units compared to the control. Similar results were obtained with minced meat products. Homayounpour et al. [46] showed that a nano-liposomal form of garlic essential oil more effectively reduces TVC and extends the shelf life of hamburgers compared to the free oil. Also, Baghi et al. [47] found that active packaging with trans-cinnamaldehyde nanoemulsions significantly slows down the growth of bacteria in minced meat, with the effectiveness also depending on the structure of the packaging system. Accordingly, Zhang et al. [43] showed that chitosan nanocapsules with caraway essential oil provided lower TVC values in lamb meat compared to free components, especially in the later stages of storage. Additionally, chitosan-based nanoparticles with incorporated *Citrus reticulata* L. essential oil reduced the presence of TVC in pork tenderloins by about 25% [48]. Incorporation of thyme and clove essential oils in chitosan–Zn nanosystems enabled a reduction in TVC to 3.75 log CFU/g and 4.20 log CFU/g in beef burgers, respectively [49]. Cellulosic NPs containing 2% propolis extract in the chitosan system were also effective, reducing the number of TVC in beef from approximately 10 to 6 log CFU/g [50].

#### 2.2.2. *Pseudomonas* spp.

*Pseudomonas* spp. represent the dominant spoilage microorganisms responsible for the deterioration of raw meat under aerobic storage conditions, as they are well adapted to low temperatures and utilize meat proteins as an energy source [51]. The results of the impact of the application of CH-OBEO and CH-OBNPs are shown in Table 3. The obtained results indicate that all chitosan coating treatments significantly (*p* < 0.05) limited the growth of *Pseudomonas* spp. after the fourth day of storage. Edible coatings may reduce oxygen availability at the meat surface, thereby altering the oxidation–reduction potential and creating less favorable conditions for aerobic microorganisms such as *Pseudomonas* spp. compared to the uncoated control [52].

At the beginning of storage, the microbial load by *Pseudomonas* spp. was similar in all samples (2.33–2.39 log CFU/g). The control sample showed a continuous increase in the number of *Pseudomonas* spp. from 2.35 log CFU/g at the beginning to 7.80 log CFU/g at day 20, thus exceeding the critical microbiological acceptability limit of 7 log CFU/g, according to The International Commission on Microbiological Specifications for Foods (ICMSF) standard [39,53]. In comparison, the CH sample reached 6.95 log CFU/g after 20 days, indicating a statistically significant (*p* < 0.05) slowdown in the growth of *Pseudomonas* spp., but not enough to prevent them from reaching almost unacceptable levels.

On the eighth day of storage, there was no statistically significant difference in the number of *Pseudomonas* spp., regardless of the treatment with free or nanoencapsulated oil and their concentrations. After 12 days of storage, a clear difference in the effect of these two forms of essential oils was observed, and on the 20th day, the number of *Pseudomonas* spp. in samples with free essential oil were 6.57, 6.58 and 6.27 log CFU/g for OBEO 0.25, 0.5 and 1% respectively—thus below the limit of 7 log CFU/g, which means that the meat was still microbiologically acceptable. This confirms that OBEO in a chitosan coating has a suppressive effect on the growth of *Pseudomonas* spp., most likely due to the presence of linalool and epi-α-cadinol, as the main components which were detected in the OBEO used as published in the study by Đorđević et al. [54]. The free form of thyme and oregano essential oils in chitosan systems showed effective inhibition of Pseudomonas spp. growth, reducing the number from 6.5 log CFU/g to almost 0.5 log CFU/g, after 30 days of storage at 4 °C [55].

An even more pronounced effect was achieved with the use of nanoencapsulated essential oil (OBNPs). The CH-OBNPs 1% treatment showed an increase in the bacterial count from 2.35 to only 5.89 log CFU/g after 20 days, while lower concentrations (0.25% and 0.5%) recorded 6.19 and 5.90 log CFU/g, respectively. This means that all CH-OBNPs treatments remained within microbiologically acceptable limits throughout the entire storage period, while the control became unacceptable between days 12 and 16.

A direct comparison of the effects of free and nanoencapsulated essential oil on the control of *Pseudomonas* spp. growth in fresh beef was presented in the study by Đorđević et al. [45], where it was found that the nanoencapsulated form of the *Satureja montana* L. essential oil provided better microbiological stability of the meat compared to the free form, which is in agreement with the results obtained in this study. Other studies also confirm that the nanoencapsulated form of an essential oil is better at reducing the number of *Pseudomonas* spp., for example, in fresh turkey filets treated with chitosan nanoparticles with incorporated essential oils of *Zataria multiflora* and *Bunium persicum*, a reduction in the number of *Pseudomonas* spp. of about 2 log CFU/g was recorded after 18 days of storage [56]. In addition, the system combining chitosan nanoparticles with the essential oil of *Satureja khuzestanica* showed an even more pronounced antimicrobial effect, reducing the final number of these bacteria by as much as 5 log units compared to the control [56]. Additionally, the application of a chitosan nanoemulsion enriched with thyme essential oil resulted in a significant reduction in the population of *Pseudomonas* spp. in fresh pork meat, where after 12 days of storage at +4 °C a reduction of approximately 20% was recorded [57]. On the other hand, in ham pieces, the addition of a nanoemulsion with the addition of *Eugenia brejoensis* essential oil at a concentration of 1% did not prove successful in reducing the number of *P. fluorescens* [58]. Other types of nanoparticles, such as Zn with the addition of propolis extract in a chitosan matrix, significantly inhibited the growth of *Pseudomonas* in beef, reducing the initial count by more than half, from 8.5 log CFU/g to almost 4 log CFU/g [50]. Chitosan nanoparticles with incorporated aloe vera gel in mutton had a statistically significant effect on reducing the number of *Pseudomonas aeruginosa* (3.68 log CFU/g), compared to the untreated sample (4.68 log CFU/g), after 12 days of refrigerated storage [59].

#### 2.2.3. Lactic Acid Bacteria

The change in the number of lactic acid bacteria (LAB) in fresh beef meat during 20 days of storage is shown in Table 4 and clearly indicates a significant influence of the applied treatments—whether the presence of the chitosan coating or the addition of free or nanoencapsulated *Ocimum basilicum* L. essential oil. Initially, the number of LAB was similar in all samples (about 1.5 log CFU/g). The control sample recorded the fastest increase—from 1.48 log CFU/g at the beginning to 6.35 log CFU/g after 20 days, which was expected because in the absence of coating and antimicrobial components, LAB grow unrestricted. The addition of CH significantly affected the reduction in the number of LAB, so from the second day to the end of storage, a statistically significant difference in their number was observed, compared to the control (*p* < 0.05).

The application of the CH-OBEO induced a concentration-dependent inhibitory effect on LAB growth. Coatings containing OBEO at 0.25% and 0.5% provided moderate reductions compared to the control; however, the highest tested concentration CH-OBEO 1% exerted higher suppression, with LAB counts remaining lower throughout the storage period. This dose-dependent response is consistent with findings reported for other essential oils incorporated into chitosan-based coatings applied to beef. Gaba et al. [55] demonstrated that chitosan films enriched with oregano and thyme essential oils at 0.5% and 1% inhibited spoilage bacteria in refrigerated beef, with the shelf life extended by approximately 10 days compared to chitosan. The relatively modest effect observed at lower OBEO concentrations in the present study may be attributed to the volatility of phenolic compounds in basil EO including linalool and eugenol which are susceptible to evaporation and interaction with meat proteins, thereby reducing their bioavailability at the meat surface when applied in free form [60].

This limitation of free EO delivery was clearly addressed by the nanoencapsulated formulations. The CH-OBNPs treatments, particularly at higher concentrations, demonstrated sustained antimicrobial activity across the entire storage period, with CH-OBNPs 1% achieving final LAB counts of 5.60 log CFU/g, lower than all free EO treatments at equivalent concentrations. This is in agreement with the previous results of the use of free and nanoencapsulated *Satureja montana* L. EO on beef stored at 4 °C for 20 days where CNP-enriched coatings exerted more pronounced and prolonged antimicrobial effects against LAB and other tested microorganisms compared to free EO at the same concentration levels [45]. The incorporation of 2% propolis extract into cellulose nanoparticles and the addition to chitosan films enabled a reduction in the LAB population in beef after 14 days of storage [50].

#### 2.2.4. Psychrotrophic Bacteria

Initial psychrotrophic bacterial counts were similar in all treatment groups, ranging from approximately 2.35 to 2.56 log CFU/g (Table 5) consistent with the expected microbiological quality of freshly processed beef 48 h post-mortem and previously reported baseline psychrotrophic counts of 2 log CFU/g on fresh meat [61]. Throughout refrigerated storage, psychrotrophic bacteria increased progressively in all samples with the most rapid and pronounced growth, reaching approximately 7.84 log CFU/g by day 20 in the control. The chitosan treatment provided a modest delay in psychrotrophic growth compared to the control, confirming the limited antimicrobial capacity of chitosan. The incorporation of OBEO into the chitosan matrix produced significantly lower psychrotrophic counts than the control or chitosan, with the inhibitory effect being dose-dependent. CH-OBEO 1% showed the most effective suppression, reaching approximately 6.85 log CFU/g at day 20, compared to 7.84 log CFU/g in the control. This is in agreement with Gaba et al. [55], who demonstrated that chitosan films enriched with 1% essential oil from oregano and thyme significantly inhibited spoilage bacteria on refrigerated beef stored for 30 days. The antimicrobial efficacy of OBEO with its major bioactive constituents, linalool, eugenol, and 1,8-cineole [54] has already been proven in meat samples [62].

CH-OBNPs demonstrated better performance compared to the corresponding CH-OBEO treatments, particularly towards the end of the storage period. At day 16, sample CH-OBNPs 1% achieved counts approximately 1.37 log CFU/g lower than the uncoated control, and by day 20, it maintained the lowest psychrotrophic count of all treatment groups (~6.67 log CFU/g). Such efficacy of the nanoparticle formulations can probably be attributed to the sustained, controlled release of bioactive compounds from the NPs, which prolongs the antimicrobial effect on the meat surface over extended storage. Moadab et al. [63] reported that chitosan coatings containing *Eryngium campestre* essential oil nanoemulsions significantly suppressed psychrotrophic counts compared to control samples after 12 days at 4 °C. A similar effect of nano-encapsulation was previously reported for beef treated with chitosan enriched with free and nanoencapsulated *Satureja montana* L. essential oil, where the application of nanoparticle-loaded coatings showed lower psychrotrophic counts throughout 20 days of chilled storage compared to essential oil treatments at equivalent concentrations [45]. The application of chitosan nanoparticles with cumin essential oil reduced the number of psychrotrophic bacteria after 7 days of storage from 6.5 log CFU/g to 6 log CFU/g in mutton, which is another confirmation of the effectiveness of chitosan nanoparticles enriched with essential oils [43]. A pronounced ability to inhibit the growth of psychrotrophic bacteria in beef was observed with the use of chitosan films with the addition of Zn nanoparticles enriched with propolis extract (2%), with approximately 4.5 log CFU/g recorded, which is half as much as the control (about 9 log CFU/g) after 14 days of storage [50].

Compared to the total viable count, it can be noticed that over time psychrotrophic bacteria consistently approached or slightly exceeded the TVC. This could be expected because of the selective advantage that psychrotrophs have under refrigeration conditions. As storage progresses, the psychrotrophs dominate the total viable population, effectively making psychrotrophic enumeration a more sensitive indicator of quality deterioration in chilled meat than total mesophilic counts alone [51].

Taken together, the data follows the antimicrobial hierarchy across the treatment groups: C < CH < CH-OBEO 0.25% ≈ CH-OBEO 0.5% < CH-OBEO 1% < CH-OBNPs 0.25% ≈ CH-OBNPs 0.5% < CH-OBNPs 1%, with statistically significant differences between groups (*p* < 0.05) at most time points. The results confirm that combining chitosan with OBEO, particularly in the nanoencapsulated form, represents an effective strategy for retarding psychrotrophic proliferation and extending the microbiological shelf life of fresh beef under aerobic refrigerated storage.

### 2.3. Changes in pH Value During Beef Storage

In fresh beef, the initial pH typically falls in the range of 5.4–5.8 as a result of post-mortem glycolysis, after which it tends to rise during refrigerated storage as spoilage microorganisms and endogenous proteolytic enzymes degrade proteins, generating alkaline volatile compounds including ammonia, biogenic amines, and other nitrogen-containing degradation products [45].

In the present study, all samples had initial pH values between 5.24 and 5.56 on day 0, consistent with expected post-rigor beef pH (Table 6). The uncoated control showed the most significant increase, reaching a pH of 7.13 by day 20, reflecting uninhibited microbial proliferation and proteolytic breakdown. The chitosan coating significantly slowed this increase (final pH 6.84), consistent with the well-established ability of chitosan to disrupt bacterial cell membranes electrostatically, thereby suppressing the growth of spoilage bacteria responsible for alkaline metabolite production [45,64].

The addition of OBEO to the chitosan matrix further reduced the rate of pH increase in a concentration-dependent manner, with CH-OBEO samples reaching final pH values of 6.65–6.71 on day 20. This can be attributed to the antimicrobial activity of eugenol, linalool, and estragole which ware identified in the OBEO [54] and proven to disrupt microbial membranes and inhibit microbial enzyme activity [65].

The CH-OBNPs treatments demonstrated comparable or better pH control relative to the CH-OBEO samples, with CH-OBNPs 1% yielding the lowest final pH of 6.30 on day 20. This advantage can be attributed to the sustained and controlled release of antimicrobial compounds from the nanoparticle matrix, which maintains effective bioactive concentrations at the meat surface over longer storage periods, compensating for the volatilization and degradation of free EO constituents [45]. Other studies have reported stable pH values during the storage of beef burger samples treated with chitosan–Zn nanoparticles enriched with thyme and clove essential oils. At the end of the storage period, no statistically significant difference in pH values was observed between the two treatments [49]. Chitosan nanoparticles incorporating 1% aloe vera gel also contributed to the maintenance of a stable pH value (6.02) after 12 days of storage in mutton samples [59].

### 2.4. Lipid Oxidation in Beef Samples During Storage

Lipid oxidation is among the primary causes of quality deterioration in fresh beef during refrigerated storage, with the TBARS assay serving as a reliable indicator of secondary oxidation products, particularly malondialdehyde (MDA), which is responsible for rancid off-odors and off-flavors [66]. Values exceeding 2 mg MDA/kg of meat are widely considered the threshold at which rancidity becomes sensorily detectable and the product is considered unacceptable to consumers [67].

All analyzed samples showed a progressive increase in TBARS values throughout the 20-day refrigerated storage period (Table 7). The uncoated control exhibited the highest degree of lipid oxidation, with TBARS values rising from 0.31 mg MDA/kg on day 0 to 3.27 mg MDA/kg by day 20. This is consistent with findings reported by Isvand et al. [68], who observed an increase in TBARS values in uncoated beef samples during refrigerated storage, confirming that unprotected fresh beef is highly susceptible to lipid peroxidation.

The chitosan coating provided only modest antioxidant protection, yielding slightly lower TBARS values than the control throughout storage (3.10 mg MDA/kg on day 20). This moderate effect is in line with the well-documented capacity of chitosan to act as a free radical scavenger and metal ion chelator, thereby partially suppressing pro-oxidant activity at the meat surface [45]. However, chitosan alone was insufficient to maintain TBARS values below the acceptability threshold by the end of the storage period, highlighting the need for the inclusion of bioactive compounds to achieve a meaningful antioxidant effect.

The incorporation of OBEO into the chitosan matrix resulted in a dose-dependent reduction in lipid oxidation across all storage intervals. At day 20, TBARS values for OB EO 0.25%, 0.5%, and 1% were 2.95, 2.25, and 2.13 mg MDA/kg, respectively—all significantly lower than both control and the chitosan coating. This antioxidant efficacy can be attributed to the complex mixture of bioactive phenolic constituents present in OBEO, probably linalool, eugenol, and 1,8-cineole, which have been shown to exhibit potent radical scavenging activity through hydrogen atom donation and metal ion chelation mechanisms [65,69]. These compounds have been detected in the applied OBEO and their antioxidant potential has been confirmed [54].

The samples treated with CH-OBNPs showed significantly better antioxidative protection compared to the corresponding concentrations of CH-OBEO, particularly at higher concentrations and longer storage times. At day 20, TBAR values for CH-OBNPs 0.25%, 0.5%, and 1% were 2.33, 2.19, and 1.97 mg MDA/kg, respectively, with the 1% NPs formulation being the only treatment that maintained TBAR below the 2 mg MDA/kg during the entire 20-day storage period. These results are consistent with the advantages of nanoencapsulation reported in the literature. As demonstrated by Moadab et al. [63] for chitosan nanoemulsions of *Eryngium campestre* EO applied to ostrich meat, nanoencapsulation can protect the volatile bioactive compounds from degradation and evaporation, significantly improving their controlled release and providing a significant antioxidant effect throughout storage. The enhanced performance of the NP formulations in the present study can be explained by the increased surface-area-to-volume ratio of nanoparticles, which improves the bioavailability of the encapsulated EO compounds within the coating matrix, facilitating a more uniform and prolonged release of antioxidant constituents at the meat surface [45]. Similarly, the application of chitosan nanoemulsions incorporated with *Schizonepeta tenuifolia* essential oil reduced TBARS values by nearly 50% in fresh pork [70].

The dose–response relationship observed for both CH-OBEO and CH-OBNPs treatments further supports the hypothesis that the antioxidant effect in this coating system can primarily be attributed to the concentration of bioactive EO constituents, rather than the chitosan matrix alone. Taken together, the TBARS results indicate that chitosan coatings enriched with BEO, particularly in their nanoencapsulated form, can represent an effective strategy of delaying lipid oxidation in fresh beef and extending its oxidative shelf life.

### 2.5. Sensory Analysis

**Figure 1:**
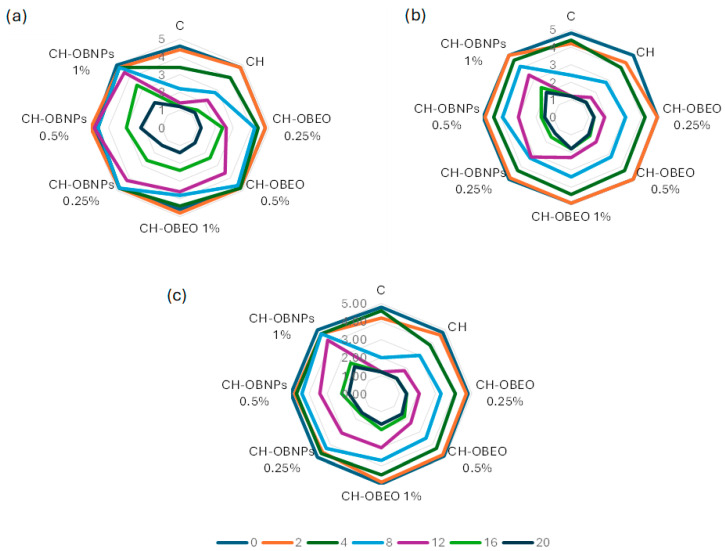
Sensory assessment of beef samples coated with chitosan and chitosan-based nanoparticles incorporating basil essential oil during storage period: (**a**) smell; (**b**) appearance; (**c**) general acceptability. C—control; CH—chitosan coating; CH-OBEO—chitosan coating enriched with *Ocimum basilicum* L. essential oil at different concentrations; CH-OBNPs—chitosan coating enriched with different concentrations of chitosan-based nanoparticles loaded with *Ocimum basilicum* L. essential oil.

Sensory quality is one of the most direct indicators of meat freshness. The sensory properties of beef samples, including odor, color, and overall acceptability, were monitored over 20 days of storage, with all treatment groups showing a progressive decline throughout the evaluation period. The initial values of all investigated parameters were highly rated regardless of the type of coating used (Figure 1). Odor values gradually declined across all groups over time as expected (Figure 1a). However, the CH-OBNPs better preserved odor scores in the second half of storage compared to the control and the chitosan coating. Similar results were already obtained in previous research with chitosan coatings enriched with *Satureja montana* L. essential oil on refrigerated beef, noting that the durability of smell was significantly increased by the application of chitosan coatings with both free and nanoencapsulated essential oil compared to the control [45]. It is notable that the application of nanoparticles significantly preserved the smell of fresh meat compared to free essential oil, especially after 12 days of storage. Free essential oil created an unnatural aroma perceived negatively by evaluators, while nanoencapsulation effectively neutralized this effect, indicating that direct oil incorporation at higher concentrations did not reflect well on smell rate at the later stages of storage.

Evaluation of color indicated that the incorporation of OBEO resulted in better values of color compared to the control or plain chitosan coating (Figure 1b). On the other hand CH-OBNPs, caused far less visual disruption and resulted in color conservation up to the day 12 of storage for the concentration of 0.25% and day 16 for higher NP concentrations. Isvand et al. [68] reported a comparable trend, finding that a chitosan coating with a nanoemulsion of *Citrus limon* essential oil had a substantial preservative effect on refrigerated beef, reducing lipid oxidation, inhibiting microbial growth, and maintaining more stable color characteristics during cold storage. The differences between the nanoincorporated and free essential oil application can be explained by the slower release of active compounds due to the presence of the biopolymer capsule.

Overall acceptability indicated that the control sample was not for use by day 8, while the chitosan coating remained at the marginal value (Figure 1c). This can be explained by the antimicrobial and antioxidative effects of chitosan itself. The samples CH-OBEO remained stable for the 12 days, with values below 3 for concentrations of 0.25 and 0.5% and a marginal value of 3 for CH-OBEO 1%. On the other hand, all CH-OBNPs samples were stable until day 16 of storage. Moadab et al. [63] reached a similar conclusion when testing chitosan coatings enriched with *Eryngium campestre* essential oil nanoemulsion on ostrich meat, reporting that odor scores decreased for every treatment across a 12-day storage period, with the control declining fastest, while chitosan coatings enriched with essential oil maintained the highest overall acceptability scores, attributed to the antimicrobial and antioxidant activities of the essential oil. Gaba et al. [55] further supported this, concluding that a chitosan coating with essential oils at 1% represents a successful strategy to improve quality and extend the shelf life of beef, with treated samples showing higher sensory retention compared to the control throughout the storage period.

## 3. Materials and Methods

### 3.1. Preparation of Chitosan Coatings

Chitosan coatings were prepared with modifications to the protocol reported by Pabast et al. [71]. A 2% chitosan solution was produced by dissolving medium molecular weight chitosan powder (Sigma-Aldrich, St. Louis, MO, USA) in 1% acetic acid (≥99.8%, Centrohem, Stara Pazova, Serbia). This dissolution was carried out using a magnetic stirrer (SCILOGEX SCI280-Pro, Rocky Hill, CT, USA) for 2 h at 95 °C. The resulting solution was filtered using filter paper (125 mm diameter, CHMLAB Group, Barcelona, Spain) to remove undissolved particles. After filtration, glycerol was added as a plasticizer at a ratio of 0.25 mL per gram of chitosan (*v*/*w*). The mixture was stirred for an additional 30 min at ambient temperature. Subsequently, Tween 60 (Sigma Aldrich, Darmstadt, Germany) (0.1%, *v*/*v*) was incorporated as an emulsifier, and the suspension was maintained at 60 °C with continuous stirring for 30 min. Upon cooling to room temperature (OBEO) or OBEO-loaded nanoparticles (OB NPs) were incorporated at 0.25%, 0.5%, or 1% (*v*/*v*) concentrations into the chitosan coating formulation and stirred for 20 min at room temperature to ensure uniform dispersion.

### 3.2. Preparation of OBEO-Loaded Chitosan Nanoparticles

OBEO was previously extracted by microwave-assisted extraction from air-dried plant material as published in Đorđević et al. [54] GC/MS and GC/FID analyses revealed the major classes of compounds: oxygen-containing monoterpenes (62.5%), sesquiterpene hydrocarbons (17.7%), oxygen-containing sesquiterpenes (11.2%) and other aromatic compounds (6.4%) [54]. The OBEO was stored at 4 °C until use.

OBNPs were prepared via ionic gelation following Zhang et al. [72]. A 0.4% chitosan solution was prepared by dispersing medium molecular weight chitosan powder (Sigma-Aldrich, St. Louis, MO, USA) in 50 mL of 1% (*v*/*v*) acetic acid with constant stirring, followed by filtration to remove insoluble matter. The filtrate’s pH was adjusted to 4.7–4.8 using 1 M NaOH. Tween 60 (0.1%, *v*/*v*) was added, and the mixture was heated to 60 °C until homogeneous. OBEO (200 mg), corresponding to a chitosan: OBEO ratio of 1:1 (*w*:*w*) dissolved in 4 mL of absolute ethanol was added dropwise to the cooled chitosan solution with stirring at 1200 rpm for 30 min. After reaching room-temperature homogenization, 30 mL of sodium tripolyphosphate solution (TPP, 1.87 mg/mL, pH 4; obtained from Centrohem, Stara Pazova, Serbia, >96% purity) was introduced gradually with stirring for 60 min, corresponding to a chitosan: TPP ratio of 3.6:1 (*w*:*w*). BEO-loaded nanoparticles (BEO-CNPs) were collected by centrifugation at 14,000 rpm for 20 min (Eppendorf 5418, Eppendorf, Hamburg, Germany) and stored at 4 °C until use.

### 3.3. Encapsulation Efficiency Determination

To assess encapsulation efficiency, 200 μL of OBNPs suspension was mixed with 5 mL of a 2 M HCl solution and heated in a boiling water bath for 30 min. After cooling, 2 mL of ethanol (96%, *v*/*v*) was added, and the mixture was centrifuged at 9000 rpm for 5 min. The supernatant’s absorbance, measured from 200 to 400 nm with a maximum at 275 nm, was recorded using a UV-Vis spectrophotometer (Cole-Parmer 2100, Cole-Parmer, Vernon Hills, IL, USA). Quantification was performed using a previously established calibration curve of OBEO in ethanol (y = 1.5171x − 0.0032, R^2^ = 0.9993). Encapsulation efficiency was calculated as follows [72]:

Encapsulation efficiency (EE, %) = (weight of OBEO loaded/weight of initial OBEO) × 100.

### 3.4. Nanoparticle Characterization

Z-average hydrodynamic diameter and polydispersity index (PDI) of unmodified CNPs and OBNPs were determined via dynamic light scattering (DLS) using a Zetasizer 7.11 instrument (Malvern Instruments, Malvern, Worcestershire, UK). Prior to analysis, samples were diluted (1:2) in distilled water to avoid multiple scattering effects and gently sonicated for 1 min to ensure proper dispersion. Measurements were performed at 25 °C using a scattering angle of 173°.

### 3.5. Preparation of Meat Samples

Raw beef tenderloin (48 h post-mortem) was procured locally, and proximate composition was determined in accordance with ISO methods: moisture (ISO 1442) [73], protein (ISO 937) [74], fat (ISO 1443) [75], and ash content (ISO 936) [76], yielding approximately: moisture about 63.0%, protein 19.73%, total fat 5.35%, and ash 1.82%. Following Lekjing et al. [77] with minor adjustments, meat surfaces (pieces of 50 ± 1 g) were subjected to surface decontamination using UV light (254 nm) on both sides for 10 min. Treated samples were immersed for 3 min in a 2% (*w*/*v*) chitosan coating containing OBEO or OBNPs, maintaining a constant sample-to-solution ratio of 10:1 (*w*/*v*). Each treatment was performed in duplicate, yielding 14 samples per treatment. Post-treatment, samples were air-dried aseptically for 15 min, placed in sterile Whirl-Pak bags, and stored at 4 ± 1 °C in a controlled incubator for 20 days. Microbiological and other analyses were conducted in triplicate at 0, 2, 4, 8, 12, 16, and 20 days. Table 8 provides a description of the various meat treatment groups.

### 3.6. Microbiological Analysis

Microbial load was determined according to standard microbiological procedures with minor modifications. Briefly, 25 g of meat was homogenized with 225 mL of sterile saline–peptone water (0.8 g/L NaCl and 1 g/L peptone) using platform shaker (Unimax 2010, Heidolph Instruments GmbH and Co. KG, Schwabach, Germany) for 15 min at medium speed. Serial decimal dilutions (10^−1^–10^−6^) were prepared in sterile diluent.

Appropriate dilutions (1 mL) were plated on selective media. Total viable count (TVC) was determined on nutrient agar (Torlak, Belgrade, Serbia) incubated at 30 °C for 48 h [78]. Psychrotrophic bacteria were enumerated after incubation at 7 °C for 7–10 days. Lactic acid bacteria (LAB) were quantified on Lactobacillus MRS agar (HiMedia Laboratories Pvt. Ltd., Mumbai, India) at 30 °C. *Pseudomonas* spp. were assessed on *Pseudomonas* CFC/CN agar (Merck & Co., Inc., Rahway, NJ, USA). After incubation, colonies were counted and reported as log CFU/g. All analyses were performed in triplicate. Since molecular or biochemical identification of enumerated bacterial strains was not performed, the numbers of LAB and *Pseudomonas* spp. should be considered presumptive, rather than definitively identified.

### 3.7. Lipid Oxidation Assessment

Lipid oxidation was quantified by the thiobarbituric acid reactive substances (TBARS) assay as described by Zhang et al. [72]. Ten grams of finely chopped meat were homogenized with 30 mL of 7.5% (*w*/*v*) trichloroacetic acid (Fisher Scientific, Loughborough, Leicestershire, UK) for 2 min at 6000 rpm using a homogenizer (TH16B, Biobase Biodustry (Shandong) Co., Ltd., Jinan, China), followed by filtration. To the filtrate, 5 mL of 20 mmol/L thiobarbituric acid (ISOLAB, Belgrade, Serbia) was added, and the mixture was incubated at 95 °C for 30 min. After cooling, absorbance at 532 nm was measured. Malondialdehyde (MDA) equivalents were quantified using a calibration curve prepared from 1,1,3,3-tetramethoxypropane (TMP) standard solutions, with a regression equation of y = 16.923x + 0.0862, R^2^ = 0.9958. Results were expressed as mg MDA/kg of meat after blank correction. All analyses were performed in triplicate.

### 3.8. pH Measurement

The pH value was determined according to ISO 2917 [79] with minor modifications. Ten grams of meat sample were homogenized with 100 mL of distilled water using a laboratory shaker (Unimax 2010, Heidolph Instruments GmbH and Co. KG, Schwabach, Germany) for 15 min at room temperature. The homogenate pH was measured directly in the mixture using a calibrated pH meter (Hanna HI 9318, Hanna Instruments, Woonsocket, RI, USA). Measurements were performed in triplicate.

### 3.9. Sensory Evaluation

Sensory evaluation was performed by seven semi-trained panelists from the Department of Food Science evaluating color, odor, and overall acceptability using a 5-point descriptive scale (5 = extremely acceptable, 1 = extremely unacceptable) [72]. A short training session was conducted prior to the sensory evaluation to ensure a consistent understanding of the assessed attributes and scoring criteria among panelists. Sensory scores were calculated as the mean of three replicate measurements obtained from independent samples. Sensory scores below 3 were considered below the acceptable level. The consistency of panel evaluations was verified by ensuring that the coefficient of variation for each attribute did not exceed 20%.

### 3.10. Statistical Analysis

Experiments were conducted in triplicate and results are presented as mean ± standard deviation. One-way ANOVA with Tukey’s post hoc test was used to compare differences, with treatments treated as the study factor in one analysis and storage days in another. Statistical significance was set at *p* < 0.05. Analyses were performed using SPSS 21.0 (IBM, Armonk, NY, USA).

## 4. Conclusions

In conclusion, chitosan-based edible coatings enriched with free and nanoencapsulated *Ocimum basilicum* L. essential oil effectively control microbial contamination in fresh beef including total viable count, lactic acid bacteria, psychrotrophic bacteria, and *Pseudomonas* spp. The incorporation of OBEO into the chitosan matrix produced a concenpsychrotrophictration-dependent increase in antimicrobial activity, while nanoencapsulated formulations outperformed free OBEO, likely due to the controlled release and improved stability of bioactive compounds within the nanoparticle matrix. These findings confirm that CH-OBNPs coatings represent a promising natural alternative to conventional preservatives for improving the microbiological safety and extending the shelf life of fresh beef. To further validate these results, further research should include the determination of the release profiles, the interaction of nanoparticles with the meat matrix and the stability of nanoparticles over a longer period of time. Taking into account the sensory evaluation, the results indicate that nanoencapsulation improves the practical use of basil essential oil-based coatings, allowing a longer effect of bioactive compounds without the deterioration of the sensory characteristics. Despite the shortcomings of the research, the obtained results support nanoencapsulation as a promising strategy for enhancing the functional efficacy of essential oil-based antimicrobial coatings in meat preservation by suppressing microbial contamination. Future research will focus on scaling up these processes and evaluating their potential industrial application.

## Figures and Tables

**Table 1:** Nanoparticle characterization parameters.

Sample	Z-Average Diameter (nm)	Polydispersity Index (PDI)	Encapsulation Efficiency (%)
CHNPs	415.4 ± 8.0	0.718	-
OBNPs	177.4 ± 8.0	0.221 ± 5.32	73.84 ± 4.63

CHNPs—chitosan nanoparticles. OBNPs—chitosan-based nanoparticles loaded with *Ocimum basilicum* L. essential oil.

**Table 2:** Changes in total viable counts (TVC) of beef samples treated by chitosan coatings containing free and nanocapsulated *Ocimum basilicum* L. essential oil during storage.

Sample	Storage Period (Day)						
	0	2	4	8	12	16	20
	log CFU/g						
C	2.35 ± 0.11 ^a,A^	2.47 ± 0.02 ^a,A^	3.60 ± 0.06 ^cd,B^	5.95 ± 0.03 ^e,C^	7.10 ± 0.08 ^e,D^	7.61 ± 0.34 ^d,E^	8.04 ± 0.06 ^e,F^
CH	2.41 ± 0.12 ^ab,A^	2.41 ± 0.01 ^a,A^	3.25 ± 0.02 ^a,B^	5.56 ± 0.04 ^d,C^	6.62 ± 0.01 ^d,D^	6.84 ± 0.17 ^c,D^	7.37 ± 0.07 ^d,E^
CH-OBEO 0.25%	2.65 ± 0.19 ^b,A^	2.46 ± 0.10 ^a,A^	3.49 ± 0.06 ^bc,B^	5.15 ± 0.04 ^c,C^	6.49 ± 0.07 ^d,D^	6.81 ± 0.07 ^c,E^	6.96 ± 0.04 ^bc,E^
CH-OBEO 0.5%	2.56 ± 0.03 ^ab,A^	3.26 ± 0.02 ^bc,B^	3.34 ± 0.05 ^ab,B^	5.09 ± 0.09 ^bc,C^	6.12 ± 0.14 ^c,D^	6.78 ± 0.02 ^c,E^	7.11 ± 0.09 ^c,F^
CH-OBEO 1%	2.56 ± 0.03 ^ab,A^	3.34 ± 0.05 ^c,B^	3.64 ± 0.06 ^cd,C^	4.21 ± 0.08 ^a,D^	5.06 ± 0.08 ^b,E^	6.52 ± 0.03 ^bc,F^	6.79 ± 0.02 ^b,G^
CH-OBNPs 0.25%	2.52 ± 0.02 ^ab,A^	3.56 ± 0.01 ^d,B^	3.81 ± 0.07 ^e,C^	4.92 ± 0.04 ^b,D^	5.12 ± 0.01 ^b,E^	6.83 ± 0.02 ^c,F^	7.12 ± 0.10 ^c,G^
CH-OBNPs 0.5%	2.57 ± 0.03 ^ab,A^	3.11 ± 0.09 ^b,B^	3.56 ± 0.03 ^cd,C^	4.18 ± 0.05 ^a,D^	4.66 ± 0.10 ^a,E^	6.19 ± 0.06 ^ab,F^	6.91 ± 0.12 ^bc,G^
CH-OBNPs 1%	2.48 ± 0.13 ^ab,A^	3.19 ± 0.05 ^bc,B^	3.71 ± 0.06 ^de,C^	4.33 ± 0.11 ^a,D^	4.88 ± 0.16 ^ab,E^	5.86 ± 0.09 ^a,F^	6.55 ± 0.10 ^a,G^

C—control; CH—chitosan coating; CH-OBEO—chitosan coating enriched with *Ocimum basilicum* L. essential oil at different concentrations; CH-OBNPs—chitosan coating enriched with different concentrations of chitosan-based nanoparticles loaded with *Ocimum basilicum* L. essential oil; Different letters indicate significant differences (*p* < 0.05) among samples in the same column (lowercase letters) and row (uppercase letters).

**Table 3:** Changes in presumptive *Pseudomonas* spp. counts in beef samples treated by chitosan coatings containing free and nanocapsulated *Ocimum basilicum* L. essential oil during storage.

Sample	Storage Period (Day)						
	0	2	4	8	12	16	20
	log CFU/g						
C	2.35 ± 0.12 ^a,A^	2.54 ± 0.01 ^b,A^	2.86 ± 0.01 ^a,B^	5.46 ± 0.01 ^b,C^	6.66 ± 0.03 ^f,D^	7.01 ± 0.10 ^e,E^	7.80 ± 0.18 ^e,F^
CH	2.38 ± 0.10 ^a,A^	2.46 ± 0.02 ^b,A^	2.76 ± 0.02 ^a,B^	4.60 ± 0.00 ^ab,C^	6.14 ± 0.01 ^e,D^	6.84 ± 0.02 ^e,E^	6.95 ± 0.04 ^d,E^
CH-OBEO 0.25%	2.38 ± 0.02 ^a,A^	2.26 ± 0.11 ^a,A^	3.30 ± 0.11 ^bc,AB^	3.65 ± 1.03 ^a,B^	5.27 ± 0.02 ^b,C^	6.24 ± 0.12 ^d,CD^	6.57 ± 0.02 ^c,D^
CH-OBEO 0.5%	2.39 ± 0.03 ^a,A^	2.53 ± 0.03 ^b,A^	3.16 ± 0.03 ^b,B^	4.43 ± 0.08 ^ab,C^	5.65 ± 0.03 ^d,D^	6.11 ± 0.10 ^cd,E^	6.58 ± 0.03 ^c,F^
CH-OBEO 1%	2.33 ± 0.06 ^a,A^	2.82 ± 0.05 ^c,B^	3.35 ± 0.04 ^c,C^	4.18 ± 0.03 ^a,D^	5.43 ± 0.09 ^c,E^	5.92 ± 0.04 ^bc,F^	6.27 ± 0.05 ^b,G^
CH-OBNPs 0.25%	2.32 ± 0.07 ^a,A^	3.05 ± 0.04 ^d,B^	3.59 ± 0.03 ^d,C^	4.57 ± 0.02 ^ab,D^	5.45 ± 0.03 ^c,E^	5.76 ± 0.02 ^b,F^	6.19 ± 0.07 ^b,G^
CH-OBNPs 0.5%	2.36 ± 0.03 ^a,A^	3.21 ± 0.08 ^de,B^	3.26 ± 0.01 ^bc,B^	3.77 ± 0.02 ^a,C^	4.57 ± 0.03 ^a,D^	5.55 ± 0.05 ^a,E^	5.90 ± 0.08 ^a,F^
CH-OBNPs 1%	2.35 ± 0.03 ^a,A^	3.37 ± 0.08 ^e,B^	3.71 ± 0.06 ^d,C^	3.94 ± 0.03 ^a,D^	5.59 ± 0.07 ^d,E^	5.82 ± 0.06 ^b,F^	5.89 ± 0.10 ^a,F^

C—control; CH—chitosan coating; CH-OBEO—chitosan coating enriched with *Ocimum basilicum* L. essential oil at different concentrations; CH-OBNPs—chitosan coating enriched with different concentrations of chitosan-based nanoparticles loaded with *Ocimum basilicum* L. essential oil; Different letters indicate significant differences (*p* < 0.05) among samples in the same column (lowercase letters) and row (uppercase letters).

**Table 4:** Changes in presumptive lactic acid bacteria (LAB) counts in beef samples treated by chitosan coatings containing free and nanocapsulated *Ocimum basilicum* L. essential oil during storage.

Sample	Storage Period (Day)						
	0	2	4	8	12	16	20
	log CFU/g						
C	1.48 ± 0.01 ^a,A^	1.78 ± 0.01 ^c,A^	2.75 ± 0.01 ^c,B^	3.44 ± 0.19 ^f,C^	5.27 ± 0.02 ^e,D^	6.38 ± 0.24 ^e,E^	6.35 ± 0.01 ^f,E^
CH	1.42 ± 0.24 ^a,A^	1.61 ± 0.06 ^a,A^	2.35 ± 0.15 ^a,B^	2.84 ± 0.05 ^bc,C^	4.92 ± 0.03 ^cd,D^	5.58 ± 0.06 ^cd,E^	5.89 ± 0.03 ^b,E^
CH-OBEO 0.25%	1.53 ± 0.02 ^a,A^	2.17 ± 0.03 ^e,B^	2.69 ± 0.08 ^bc,C^	3.01 ± 0.10 ^cd,D^	5.06 ± 0.09 ^d,E^	5.58 ± 0.05 ^cd,F^	6.28 ± 0.06 ^ef,G^
CH-OBEO 0.5%	1.54 ± 0.04 ^a,A^	1.74 ± 0.04 ^bc,A^	2.44 ± 0.19 ^ab,B^	3.33 ± 0.07 ^ef,C^	4.50 ± 0.07 ^b,D^	5.25 ± 0.12 ^bc,E^	6.30 ± 0.08 ^ef,F^
CH-OBEO 1%	1.53 ± 0.06 ^a,A^	1.62 ± 0.09 ^ab,A^	2.34 ± 0.03 ^a,B^	2.76 ± 0.05 ^ab,C^	3.66 ± 0.05 ^a,D^	4.46 ± 0.17 ^a,E^	5.97 ± 0.04 ^bc,F^
CH-OBNPs 0.25%	1.55 ± 0.04 ^a,A^	1.82 ± 0.04 ^c,B^	2.71 ± 0.06 ^c,C^	3.34 ± 0.0 ^ef,D^	4.87 ± 0.05 ^c,E^	5.68 ± 0.05 ^d,F^	6.17 ± 0.07 ^de,G^
CH-OBNPs 0.5%	1.52 ± 0.06 ^a,A^	2.23 ± 0.03 ^e,B^	2.84 ± 0.05 ^c,C^	3.18 ± 0.05 ^de,D^	4.55 ± 0.04 ^b,E^	5.53 ± 0.05 ^cd,F^	6.07 ± 0.05 ^cd,G^
CH-OBNPs 1%	1.55 ± 0.02 ^a,A^	1.97 ± 0.02 ^d,B^	2.36 ± 0.03 ^a,C^	2.56 ± 0.04 ^a,D^	4.43 ± 0.02 ^b,E^	5.15 ± 0.04 ^b,F^	5.60 ± 0.07 ^a,G^

C—control; CH—chitosan coating; CH-OBEO—chitosan coating enriched with *Ocimum basilicum* L. essential oil at different concentrations; CH-OBNPs—chitosan coating enriched with different concentrations of chitosan-based nanoparticles loaded with *Ocimum basilicum* L. essential oil; Different letters indicate significant differences (*p* < 0.05) among samples in the same column (lowercase letters) and row (uppercase letters).

**Table 5:** Changes in psychrotrophic bacteria counts in beef samples treated by chitosan coatings containing free and nanocapsulated *Ocimum basilicum* L. essential oil during storage.

Sample	Storage Period (Day)						
	0	2	4	8	12	16	20
	log CFU/g						
C	2.35 ± 0.09 ^a,A^	2.95 ± 0.03 ^d,B^	3.34 ± 0.02 ^b,C^	5.67 ± 0.03 ^f,D^	6.98 ± 0.0 ^f,E^	7.13 ± 0.03 ^e,F^	7.84 ± 0.04 ^g,G^
CH	2.47 ± 0.08 ^ab,A^	2.75 ± 0.03 ^bc,B^	3.22 ± 0.00 ^ab,C^	5.22 ± 0.01 ^e,D^	6.76 ± 0.04 ^e,E^	6.89 ± 0.01 ^d,F^	7.28 ± 0.05 ^f,G^
CH-OBEO 0.25%	2.55 ± 0.01 ^b,A^	2.81 ± 0.04 ^c,B^	3.34 ± 0.03 ^ab,C^	5.02 ± 0.12 ^d,D^	5.97 ± 0.02 ^d,E^	6.55 ± 0.03 ^bc,F^	6.89 ± 0.04 ^de,G^
CH-OBEO 0.5%	2.55 ± 0.03 ^b,A^	2.76 ± 0.03 ^bc,B^	3.15 ± 0.03 ^a,C^	4.66 ± 0.04 ^c,D^	5.55 ± 0.02 ^b,E^	6.59 ±0.07 ^c,F^	6.95± 0.01 ^e,G^
CH-OBEO 1%	2.49 ± 0.02 ^b,A^	2.79 ± 0.09 ^bc,B^	3.05 ± 0.02 ^a,C^	4.52 ± 0.03 ^c,D^	5.37 ± 0.01 ^a,E^	6.46 ± 0.04 ^bc,F^	6.85 ± 0.04 ^cd,G^
CH-OBNPs 0.25%	2.56 ± 0.01 ^b,A^	2.66 ± 0.03 ^ab,B^	3.11 ± 0.01 ^a,C^	3.84 ± 0.06 ^a,D^	5.75 ± 0.05 ^c,E^	6.55 ± 0.04 ^bc,F^	6.79 ± 0.02 ^bc,G^
CH-OBNPs 0.5%	2.50 ± 0.02 ^b,A^	2.55 ± 0.03 ^a,A^	3.20 ± 0.07 ^ab,B^	3.69 ± 0.09 ^a,C^	5.63 ± 0.01 ^b,D^	6.36 ± 0.03 ^b,E^	6.75 ± 0.03 ^ab,F^
CH-OBNPs 1%	2.56 ± 0.03 ^b,A^	2.60 ± 0.06 ^a,A^	3.75 ± 0.15 ^c,B^	4.12 ± 0.01 ^b,C^	5.34 ± 0.02 ^a,D^	5.76 ± 0.18 ^a,E^	6.67 ± 0.02 ^a,F^

C—control; CH—chitosan coating; CH-OBEO—chitosan coating enriched with *Ocimum basilicum* L. essential oil at different concentrations; CH-OBNPs—chitosan coating enriched with different concentrations of chitosan-based nanoparticles loaded with *Ocimum basilicum* L. essential oil; Different letters indicate significant differences (*p* < 0.05) among samples in the same column (lowercase letters) and row (uppercase letters).

**Table 6:** pH changes in beef samples treated with chitosan coatings containing free and nanocapsulated *Ocimum basilicum* L. essential oil during storage.

Sample	Storage Period (Day)						
	0	2	4	8	12	16	20
C	5.49 ± 0.07 ^b,A^	5.59 ± 0.08 ^a,A^	5.84 ± 0.07 ^a,B^	6.11 ± 0.02 ^a,C^	6.84 ± 0.03 ^d,D^	6.94 ± 0.06 ^e,D^	7.13 ± 0.02 ^f,E^
CH	5.24 ± 0.10 ^a,A^	5.57 ± 0.06 ^a,B^	5.73 ± 0.05 ^a,BC^	5.98 ± 0.03 ^a,CD^	6.26 ± 0.06 ^c,D^	6.77 ± 0.22 ^de,E^	6.84 ± 0.07 ^c,E^
CH-OBEO 0.25%	5.52 ± 0.03 ^b,A^	5.58 ± 0.02 ^a,A^	5.59 ± 0.03 ^a,A^	5.93 ± 0.10 ^a,B^	6.07 ± 0.05 ^b,B^	6.51 ± 0.06 ^bc,C^	6.65 ± 0.05 ^bc,C^
CH-OBEO 0.5%	5.53 ± 0.04 ^b,A^	5.59 ± 0.03 ^a,A^	5.58 ± 0.17 ^a,A^	5.86 ± 0.16 ^a,B^	6.11 ± 0.26 ^b,C^	6.57 ± 0.09 ^cd,D^	6.71 ± 0.04 ^cd,D^
CH-OBEO 1%	5.56 ± 0.03 ^b,A^	5.61 ± 0.03 ^a,AB^	5.66 ± 0.01 ^a,B^	5.80 ± 0.01 ^a,C^	6.07 ± 0.04 ^c,D^	6.51 ± 0.06 ^cd,E^	6.65 ± 0.02 ^de,F^
CH-OBNPs 0.25%	5.52 ± 0.04 ^b,A^	5.69 ± 0.03 ^a,A^	5.67 ± 0.02 ^a,B^	6.16 ± 0.55 ^a,C^	6.11 ± 0.03 ^b,D^	6.35 ± 0.05 ^abc,E^	6.69 ± 0.02 ^cd,F^
CH-OBNPs 0.5%	5.49 ± 0.04 ^b,A^	5.57 ± 0.03 ^a,A^	5.72 ± 0.03 ^a,B^	5.85 ± 0.07 ^a,C^	6.11 ± 0.02 ^b,D^	6.26 ± 0.04 ^ab,E^	6.57 ± 0.03 ^b,F^
CH-OBNPs 1%	5.55 ± 0.05 ^b,A^	5.91 ± 0.58 ^a,A^	5.97 ± 0.59 ^a,A^	6.41 ± 1.16 ^a,A^	5.92 ± 0.03 ^a,A^	6.18 ± 0.03 ^a,A^	6.30 ± 0.06 ^a,A^

C—control; CH—chitosan coating; CH-OBEO—chitosan coating enriched with *Ocimum basilicum* L. essential oil at different concentrations; CH-OBNPs—chitosan coating enriched with different concentrations of chitosan-based nanoparticles loaded with *Ocimum basilicum* L. essential oil; Different letters indicate significant differences (*p* < 0.05) among samples in the same column (lowercase letters) and row (uppercase letters).

**Table 7:** Malondialdehyde (MDA) content in beef samples treated with chitosan coatings containing free and nanocapsulated *Ocimum basilicum* L. essential oil during storage.

Sample	Storage Period (Day)						
	0	2	4	8	12	16	20
	mg/kg						
C	0.31 ± 0.04 ^a,A^	0.77 ± 0.18 ^b,AB^	0.91 ± 0.09 ^c,B^	1.35 ± 0.06 ^d,BC^	1.60 ± 0.06 ^d,C^	1.70 ± 0.09 ^cd,C^	3.27 ± 0.52 ^b,D^
CH	0.29 ± 0.08 ^a,A^	0.67 ± 0.07 ^ab,B^	0.81 ± 0.10 ^bc,BC^	0.95 ± 0.03 ^c,BC^	1.05 ± 0.07 ^c,C^	0.98 ± 0.11 ^a,BC^	3.10 ± 0.26 ^b,D^
CH-OBEO 0.25%	0.28 ± 0.02 ^a,A^	0.62 ± 0.01 ^ab,B^	0.67 ± 0.04 ^ab,BC^	0.80 ± 0.06 ^b,C^	0.95 ± 0.06 ^bc,D^	2.11 ± 0.10 ^e,E^	2.95 ± 0.04 ^b,F^
CH-OBEO 0.5%	0.30 ± 0.01 ^a,A^	0.50 ± 0.01 ^a,B^	0.55 ± 0.00 ^a,C^	0.80 ± 0.01 ^b,D^	0.85 ± 0.00 ^ab,E^	1.57 ± 0.02 ^c,F^	2.25 ± 0.03 ^a,G^
CH-OBEO 1%	0.30 ± 0.02 ^a,A^	0.48 ± 0.00 ^a,B^	0.54 ± 0.03 ^a,C^	0.75 ± 0.04 ^ab,D^	0.76 ± 0.01 ^a,D^	1.86 ± 0.02 ^d,E^	2.13 ± 0.02 ^a,F^
CH-OBNPs 0.25%	0.28 ± 0.01 ^a,A^	0.53 ± e0.01 ^a,B^	0.56 ± 0.04 ^a,B^	0.65 ± 0.04 ^a,C^	0.83 ± 0.01 ^a,D^	1.02 ± 0.02 ^a,E^	2.33 ± 0.03 ^a,F^
CH-OBNPs 0.5%	0.29 ± 0.01 ^a,A^	0.54 ± 0.03 ^a,B^	0.61 ± 0.01 ^a,B^	0.70 ± 0.01 ^ab,C^	0.78 ± 0.02 ^a,D^	0.96 ± 0.04 ^a,E^	2.19 ± 0.05 ^a,F^
CH-OBNPs 1%	0.32 ± 0.01 ^a,A^	0.48 ± 0.05 ^a,B^	0.56 ± 0.03 ^a,B^	0.66 ± 0.04 ^a,C^	0.84 ± 0.05 ^ab,D^	1.34 ± 0.02 ^b,E^	1.97 ± 0.02 ^a,F^

C—control; CH—chitosan coating; CH-OBEO—chitosan coating enriched with *Ocimum basilicum* L. essential oil at different concentrations; CH-OBNPs—chitosan coating enriched with different concentrations of chitosan-based nanoparticles loaded with *Ocimum basilicum* L. essential oil; Different letters indicate significant differences (*p* < 0.05) among samples in the same column (lowercase letters) and row (uppercase letters).

**Table 8:** Description of the samples used in the research.

Coating	Description
C	Control sample
CH	Beef coated with chitosan
CH-OBEO 0.25%	Beef coated with chitosan with the addition of 0.25% OBEO
CH-OBEO 0.5%	Beef coated with chitosan with the addition of 0.5% OBEO
CH-OBEO 1%	Beef coated with chitosan with the addition of 1% OBEO
CH-OBNPs 0.25%	Beef coated with chitosan with the addition of 0.25% OBNPs
CH-OBNPs 0.5%	Beef coated with chitosan with the addition of 0.5% OBNPs
CH-OBNPs 1%	Beef coated with chitosan with the addition of 1% OBNPs

## Data Availability

Data are available from the corresponding author on request.
